# Arsenic, Cadmium, Lead, and Mercury in Sweat: A Systematic Review

**DOI:** 10.1155/2012/184745

**Published:** 2012-02-22

**Authors:** Margaret E. Sears, Kathleen J. Kerr, Riina I. Bray

**Affiliations:** ^1^Children's Hospital of Eastern Ontario Research Institute, Ottawa, ON, Canada K1H 8L1; ^2^Clinical Epidemiology, Ottawa Hospital Research Institute, Ottawa, ON, Canada K1Y 4E9; ^3^Environmental Health Clinic, Women's College Hospital, Toronto, ON, Canada M5S 1B2; ^4^Department of Family and Community Medicine, University of Toronto, Toronto, ON, Canada M5T 1W7

## Abstract

Arsenic, cadmium, lead, and mercury exposures are ubiquitous. These toxic elements have no physiological benefits, engendering interest in minimizing body burden. The physiological process of sweating has long been regarded as “cleansing” and of low risk. Reports of toxicant levels in sweat were sought in Medline, Embase, Toxline, Biosis, and AMED as well as reference lists and grey literature, from inception to March 22, 2011. Of 122 records identified, 24 were included in evidence synthesis. Populations, and sweat collection methods and concentrations varied widely. In individuals with higher exposure or body burden, sweat generally exceeded plasma or urine concentrations, and dermal could match or surpass urinary daily excretion. Arsenic dermal excretion was severalfold higher in arsenic-exposed individuals than in unexposed controls. Cadmium was more concentrated in sweat than in blood plasma. Sweat lead was associated with high-molecular-weight molecules, and in an interventional study, levels were higher with endurance compared with intensive exercise. Mercury levels normalized with repeated saunas in a case report. Sweating deserves consideration for toxic element detoxification. Research including appropriately sized trials is needed to establish safe, effective therapeutic protocols.

## 1. Introduction

No person is without some level of toxic metals in their bodies, circulating and accumulating with acute and chronic lifetime exposures. An individual may take numerous measures to minimize exposures and to optimize metabolism and excretion of toxic elements in the stool and urine with diet, supplements, and chelation therapy [1, 2]; however, an often overlooked route of excretion of toxicants is via the process of sweating [3].

Sweating with heat and/or exercise has been viewed throughout the ages, by groups worldwide, as “cleansing.” As part of a scoping review regarding arsenic, cadmium, lead, and mercury, we reviewed the scientific literature pertaining to toxicant excretion in sweat.

### 1.1. Arsenic, Cadmium, Lead, and Mercury: Background

While many chemical elements are essential for life, arsenic, cadmium, lead, and mercury have no known beneficial effect in humans. On the contrary, all four elements are confirmed or probable carcinogens, and they exhibit wide-ranging toxic effects on many bodily systems, including the nervous, endocrine, renal, musculoskeletal, immunological, and cardiovascular systems [4–7].

Children and the fetus are most at risk of harm, with early exposures potentially predisposing the youngster over his/her lifetime to multisystem ailments, as well as lower IQ and dysfunctional behavior. In older populations there is increased likelihood of early cognitive decline, as well as a range of conditions including kidney and cardiovascular disease, diabetes, and osteoporosis [4–7].

Some populations are exposed to elevated levels of toxic elements by virtue of geochemistry, resulting in groundwater or foods with elevated levels of toxic elements (e.g., elevated arsenic in groundwater, most famously in parts of Asia such as Bangladesh but also elsewhere; cadmium that accumulates in foods grown in particular locations with high levels in soils or from fertilizers, including shellfish [8], grains [9], and brassicas [10]; and mercury in fish and seafoods). Tobacco avidly accumulates cadmium and lead from soil, making smoking a major source of exposure. In addition, valuable and unique properties of arsenic, cadmium, lead, and mercury have made them integral in many products, including electronics, batteries, and alloys. Modern environmental exposures arise from mining, refining, and industrial processes (e.g., arsenic from precious metal mining and refining, mercury from chloralkali production, or lead and cadmium from mining, refining, and recycling these and other metals such as zinc); the vestiges of older products (e.g., pesticides, leaded gasoline, paint and plumbing, mercury-containing switches and thermometers, and arsenical wood preservatives); ongoing uses (e.g., arsenical veterinary drugs, and mercury-containing dental amalgams, preservatives, and lamps); as well as emissions from burning coal and other incineration (including cremation).

With toxic elements ubiquitous in our air, water, food, and the physical environment, as well as in many consumer products, prudent avoidance is not always possible. Although signs and symptoms of chronic disease are consistent with effects of arsenic, cadmium, lead, and/or mercury, physicians commonly have a low index of clinical suspicion, and therefore levels of toxic elements are seldom investigated. Diagnosis may be challenging because multiple chemicals may contribute to subtle effects in chronic illnesses of an individual, and the effects may be synergistic. A recent review called for mercury assessment in all patients presenting with hypertension or any vascular disease [11], but other toxic elements such as lead [12] may also be implicated at levels commonly observed in the population. “Interaction Profiles” [13] compiled by the US Agency for Toxic Substances and Disease Registry report that renal toxicities of mixtures of lead plus mercury are greater than would be predicted knowing the toxicity dose response of the individual elements. Similarly, neurological toxicities of mixtures of lead plus arsenic, lead plus methylmercury, and lead plus cadmium are supra-additive [13].

### 1.2. Sweating: Background

Increasing the thermal load on the body activates heat loss mechanisms including increased circulation throughout the skin and sweating [14], with blood flow to the skin increasing from a baseline of 5–10%, to 60–70% of the cardiac output [15]. Maximal sweating occurs within 15 minutes and the fluid loss may be as high as 2 L/h in an “acclimatized” person who regularly sweats [16].

Eccrine sweat is produced in tubular coil glands under the skin surface in response to heat and, or work stress. Capillaries as well as adjacent adipose tissue may contribute to secretions from sebaceous and apocrine glands, as has been seen in research using sweat patches to detect drugs of abuse [17]. Sweat arises from the blood supply to the sweat gland, but is not simply an ultrafiltrate of blood plasma; sodium and chloride are lower in sweat than in serum, as salt loss is restricted by reabsorption in the gland [18]. Both the concentration and total loss of salt (sodium chloride) in sweat vary widely among individuals [19], as well as with acclimatization to exercise and heat [20]. In an early study, Robinson et al. demonstrated that with serum salt depletion the kidneys responded within hours by restricting excretion into the urine, while the sweat glands responded only after days with decreased concentrations in the sweat [21]. Potassium, urea, ammonia, and lactic acid concentrations are higher in sweat than in plasma, although these levels are also regulated to some extent by reabsorption in the ductal tubule of the sweat gland [22]. In one study of successive exercise sessions with cool-down breaks, over the short-term sodium, potassium, calcium, and magnesium excretion in sweat remained constant, while zinc excretion fell [23]. It is unclear whether reabsorption or depletion of plasma supply resulted in diminishing zinc losses.

Children, with greater surface area in comparison to body mass, have been observed in research studies to sweat less than adults, with sweating increasing through puberty [24]. Although some research has indicated that children's thermoregulation and heat tolerance may be less robust than adults, these findings may be at least in part an artifact of study designs and models for interpretation [25]. In research involving exercise and heat, it may be a challenge to maintain ongoing, consistent motivation among children.

## 2. Methods

### 2.1. Search Strategy

Medline, Embase, Toxline, Biosis, and AMED were searched, with no restriction on date or language, to March 22, 2011. These records were supplemented with searches for other research by key authors, searches of citations and reference lists of key reports, and “related articles.”

Neither sweating nor toxic elements are exclusively modern topics of research, so in order to search older literature for all chemical forms, the online version of the Chemical Rubber Company Handbook was searched for all arsenic, cadmium, lead, and mercury compounds, and lists of keywords were extracted from these lists. Searches using these keywords yielded records that were not identified in searches using the four chemical abstracts service (CAS) numbers or the medical subject headings (MeSHs) for arsenic, cadmium, lead, and mercury. CAS numbers and MeSHs are intended for specific individual chemicals or records referring to unspecified compounds—the tool cannot simultaneously be both specific and general. Toxic element records were searched for terms related to sweating, perspiration, sauna, steam baths, exercise, depuration, and secretion or excretion from skin. Bibliographic records were imported, duplicates were removed, and reports were screened using Zotero 2.03 (http://www.zotero.org/).

### 2.2. Report Screening and Inclusion

Titles and abstracts were screened by one investigator (MS), for primary reports with data on one or more of the toxic elements in sweat, with at least a substantial abstract in English. Reviews were included at this level, to search reference lists. Two investigators (MS and KK) independently screened studies for inclusion, and extracted and verified data. All studies presenting quantitative human data on levels of arsenic, cadmium, lead, and/or mercury were included, regardless of experimental design, or methods of sweat collection or chemical analysis.

## 3. Results

**Figure 1:**
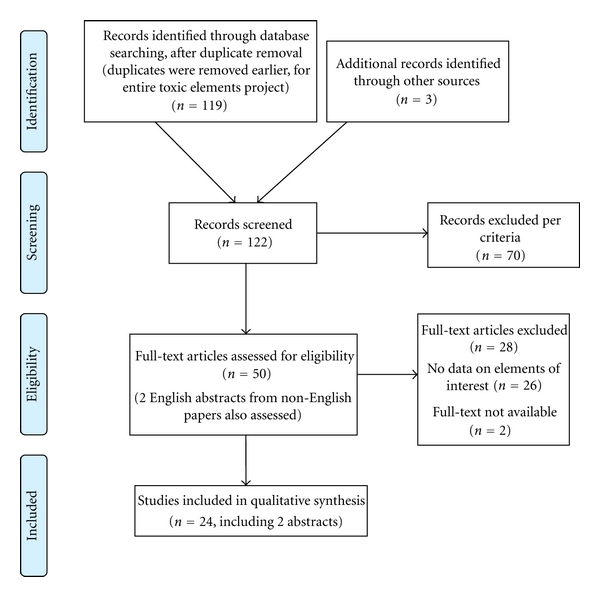
PRISMA flow diagram of evidence searches and inclusion.

Of 122 bibliographic records identified, 70 did not meet inclusion criteria at first screening, 52 full-text articles were sought for full-text screening, and 50 were obtained and screened. Data from the extended abstract of a report in German [26] and the conclusion from the abstract of one report in Russian [27] that were not obtained in full text were noted. Twenty-four reports of 22 or 23 trials or studies (it is unclear if two studies from one institution reported results twice for a subset of participants [22, 28]) were included in evidence synthesis. Searching, screening, and study inclusion are summarized in the modified PRISMA flow diagram, Figure 1.

### 3.1. Excretion of Toxic Elements in Sweat

Along with essential minerals, sweat is an acknowledged excretory route for toxic metals. For instance, it is recommended to sample hair close to the scalp because content of toxic elements may be elevated along the shaft, from either environmental contamination or excreted toxins in sweat and sebum [32, 42]. The minerals generally arise from blood serum [28], with contribution from dermally absorbed occupational exposures, which might not be reflected in blood or urine [35, 37]. Sweating was induced by sauna, exercise, or pilocarpine iontophoresis to measure the concentration of the heavy metals in the sweat, while sauna and exercise were used for therapy. Study participants included workers with occupational exposures and individuals with no occupational exposures who were well or experiencing chronic ill health, and in two studies participants were intentionally dosed with lead [34, 37]. Studies that have examined the presence of toxic metals in sweat are summarized in Tables 1, 2, 3, and 4, for arsenic, cadmium, lead, and mercury, respectively.

*Arsenic* accumulates highly in the skin, and causes characteristic skin lesions, but little information is available on levels in sweat. Yousuf et al. recently found that excretion of arsenic was greatest from the skin of patients with skin lesions, slightly but not statistically significantly lower from arsenic-exposed controls, and severalfold lower from nonexposed controls [29]. Genuis et al. measured numerous toxic elements in blood plasma, urine, and sweat of 20 study subjects (10 healthy and 10 with chronic health problems) [3]. The maximum sweat arsenic concentration was 22 *μ*g/L. On average, arsenic was 1.5-fold (in males) to 3-fold (in females) higher in sweat than in blood plasma; however, arsenic was excreted at lower concentrations in sweat than in urine [3].

*Cadmium* in sweat was examined in six studies [3, 22, 28, 30–33], with concentrations in sweat ranging from <0.5–10 *μ*g/L [28] to 0.36–35.8 *μ*g/L [3]. Stauber and Florence concluded that sweat may be an important route for excretion of cadmium when an individual is exposed to high levels [22, 28], a finding that was confirmed by observing that the total daily excretion of cadmium was greater in sweat than in urine [3, 32]. The maximum cadmium concentration observed in sweat was 35.8 *μ*g/L [3].

*Lead* was examined in eleven studies [3, 22, 26–28, 33–38]. In 1973, Hohnadel et al. suggested that “sauna bathing might provide a therapeutic method to increase elimination of toxic trace metals” [38]. In two males, 36% and 50% of sweat lead was of molecular weight > 30,000, as measured by ultrafiltration, suggesting excretion of organic complexes rather than simple ions [22]. Lead excretion was lower in females taking birth control medications compared with females not taking medications, or males [28]. Haber et al. found that prolonged endurance workouts (rowing) ameliorated elevated blood lead levels in exposed workers but did not alter levels in control subjects and did not affect urine levels [26]. They suggested that the elimination route was not urine, but potentially sweat or/and bile. Omokhodion and Crockford carried out several studies of trace elements in sweat, including a study of lead ingestion by two human participants [34]. Sweat lead levels did not increase immediately with elevated blood lead, although the authors make reference to an older study with longer followup wherein lead in underarm pads doubled in the five days following ingestion. Omokhodion and Howard also reported higher lead in sweat of exposed workers compared with unexposed controls [35], and in another study that sweat and blood lead levels were the only two variables that correlated among blood, urine, sweat, and saliva [36]. The English abstract of a 1991 case report in Russian indicated that sauna increased excretion of toxic elements and resulted in clinical improvements [27]. Sweat lead levels up to 283 *μ*g/L have been observed in nonoccupationally exposed subjects [38] and up to 17,700 *μ*g/L in workers [37], where it is noted that lead in sweat may partially originate from material absorbed within the skin that was not removed by pretest cleaning protocols [35]. Indeed, although dermal application of lead via hair follicles, sweat ducts, and diffusion does not result in immediate increases in blood or urine lead concentrations, dermal absorption was demonstrated using the Pb-204 isotope [43], lead powder, and salt [37].

*Mercury*. In 1973, Lovejoy et al. noted that exposure to mercury does not always correlate with urine mercury levels and that elimination by other routes such as sweat may be an explanation [41]. They suggested, “sweating should be the initial and preferred treatment of patients with elevated mercury urine levels.” In a 1978 case report, a severely poisoned worker was rescued with chelation therapy, followed by a regimen of daily sweat and physiotherapy over several months during which the sweat mercury level returned to normal and the patient recovered [40]. Robinson measured mercury in sweat repeatedly in two volunteers, observing sweat to urine concentration ratios ranging from less than 0.1 to greater than 5. Sweat mercury concentrations varied widely from day to day, and there was no correlation with urine levels. Sweat mercury levels of 1.5 *μ*g/L were observed by Genuis et al. [3] and 1.4 *μ*g/L by Robinson and Skelly [39].

## 4. Discussion

Arsenic, cadmium, lead, and mercury may be excreted in appreciable quantities through the skin, and rates of excretion were reported to match or even exceed urinary excretion in a 24-hour period. This is of particular interest should renal compromise limit urinary excretion of toxic elements.

Most of the research identified was over 20 years old, and collection methods varied widely. Although authors described thorough precleaning methods, sweat concentrations measured in research settings are not well validated and varied according to the location on the body, collection method, and from day to day according to other variables such as hydration. Sweat contains metals not only from the blood plasma, but also evidently originating from dermal layers (particularly with significant dermal exposures, as for workers in welding, smelting, or battery manufacturing). It would appear that large variabilities in measured concentrations, apart from collection methods as mentioned above, were likely the result of differences in excretion amongst widely varying individuals with ranges of body burdens, genetic polymorphisms affecting detoxification efficiency, and physiological states, coupled with necessarily crude if simple experimental techniques. These variations were very much greater than would be expected due to limitations of analytical methods. Although analytical methods have improved over the years, analysis of these metals was commonplace at the time of the studies. Authors generally reported analytical methods rigorously or provided references to thorough descriptions and included internal standards and some indication of sensitivity.

The observation that between a third and a half of lead in sweat may be associated with high-molecular-weight molecules [22] merits replication, including examination of additional toxic elements and characterization of the associated molecules previously observed. Excretion of these large molecules also suggests that sweating may be a means of excretion of metals complexed with natural or synthetic chelating agents.

Yousuf et al.'s recent study demonstrating a 2 : 1 molar ratio of zinc : arsenic and increased vitamin E in skin secretions suggests potential therapeutic supplementation to accommodate these biochemical requirements. Vitamin E, zinc, and other nutrients are required for methylation and detoxification of arsenic within the body, and vitamin E supplementation improves the skin manifestations in arsenicosis [29].

From an occupational health perspective, lead, and presumably other toxic elements, may be absorbed via the skin, which supports showering at work and further suggests the possibility of purging workers' skin by washing with a chelating agent (e.g., EDTA rinses extracted lead from workers' skin in methods validation experimentation [38]). It is unknown if sweating during the workday may affect dermal absorption, or if forced sweating at the end of the workday would be beneficial. It is also unknown if increased blood flow to the skin could possibly enhance absorption into the bloodstream, or if worker health could be optimized by a combination of workplace skin cleaning and sweating interventions.

Sweating has long been perceived to promote health, not only accompanying exercise but also with heat. Worldwide traditions and customs include Roman baths, Aboriginal sweat lodges, Scandinavian saunas (dry heat; relative humidity from 40% to 60%), and Turkish baths (with steam). Infrared saunas heat exposed tissues with infrared radiation, while air temperatures remain cooler than in other saunas.

Sweating is a long-standing, if recently forgotten, aspect of mercury detoxification. Various strategies used to maintain the mercury mining workforce have been explored over the centuries. In Spain and colonies, long the western world's primary sources of mercury, sending ill workers to warmer climes away from the exposure to drink weak beer (the hydrogen peroxide catalase oxidation of elemental mercury to ionic mercury is competitively inhibited by alcohol, increasing mercury in exhaled breath [44]) and to work in the heat (presumably to sweat out the “vapors”) was a common and effective strategy centuries ago; tremors, salivation, and mouth ulcers resolved generally within a few weeks [45].

With acclimatization and regular use, the sauna is generally well tolerated by all ages [46], though medical supervision may be recommended during initial sessions for children, the elderly, or those with compromised health. Varying qualities of evidence indicate potential short- and long-term improvements for cardiovascular, rheumatological and respiratory conditions; contraindications include unstable angina pectoris, recent myocardial infarction, severe aortic stenosis, and high-risk pregnancy [15, 46]. Sweating is not only observed to enhance excretion of the toxic elements of interest in this paper, but also may increase excretion of diverse toxicants, as observed in New York rescue workers [47], or in particular persistent flame retardants [48] and bisphenol-A [49].

Optimizing the potential of sweating as a therapeutic excretory mechanism merits further research. To date, the large body of research into homeostasis of the most common metals (sodium, potassium, and to a lesser extent, magnesium, calcium, and zinc) and conditioning or adaptation to regular sweating by athletes has not been matched with studies of excretion of trace elements. Limited research suggests indirectly that conditioning may not restrict excretion of nonessential elements. Combination therapies, such as administration of *n*-acetyl cysteine, vitamin C, a chelating agent, or low doses of ethanol (for mercury), to name a few possibilities, along with sauna and/or exercise therapy to induce sweating, may be fruitful avenues of investigation.

It has been noted that among people whose health is compromised by toxicants, heat regulatory mechanisms of the autonomic nervous system are often affected, resulting in a failure to sweat readily [3]. In these cases, along with diet and nutritional supplementation to remediate biochemical imbalances, interventions to consider include brushing the skin, niacin to assist with vasodilation, and exercise prior to sauna use [50]. Clinical experience is that with persistence and ample hydration patients do eventually start to sweat. This is often a sign that the autonomic nervous system function is beginning to improve. With enhanced ability to sweat, detoxification is facilitated, which can ultimately result in clinical improvement.

For biomonitoring and research purposes, modern validated methods are desirable to collect and measure elements in sweat, so this means of excretion may be considered in the context of other measures such as urine, blood, feces, and hair concentrations. Considerations for dry and wet collection methods were recently discussed in the context of essential solutes [51, 52].

Undoubtedly further research in this area would improve understanding, but the available evidence suggests that physicians could consider recommending sweating as tolerated via exercise (preferred) and/or use of a sauna as a low-risk, potentially beneficial treatment for individuals who may be experiencing effects of toxic elements, or for individuals with regular exposure to or accretion of toxicants.

## 5. Conclusions

Sweating offers potential and deserves consideration, to assist with removal of toxic elements from the body. As toxic elements are implicated in many serious chronic conditions, research is needed in patients with select conditions to evaluate the body burden and to test the efficacy of source removal, dietary choices and supplements, interventions that induce sweating, and treatments with drugs, all to enhance excretion of toxic elements with the goal of clinical improvement. There is a clear need for robust trials, appropriately sized to assess clinical outcomes, from which therapeutic protocols can be derived. Both biochemical and clinical outcomes should be examined in order to develop and monitor clinical interventions that are both safe and effective.

## Figures and Tables

**Table 1:** Studies of excretion of arsenic in sweat.

Study	Country, participants	Study design and intervention	Key findings (concentrations of *μ*g/L unless otherwise indicated)
Yousuf et al. 2011 [29]	Bangladesh 20 arsenicosis patients with melanosis and leucomelanosis 20 controls with As in drinking water 20 unexposed controls	Secretions from chest, back, and abdomen collected for 24 h, on gauze pads (8-fold; 2 × 3 inches) attached to fitted T-shirt	As secretion severalfold greater for As-exposed groups No significant difference between patients and As-exposed controls 2 zinc atoms excreted per As atom Vitamin E excreted with As

Genuis et al. 2010 [3]	Canada 10 with chronic conditions 10 healthy	Simultaneous measurement of As in blood plasma, urine, and sweat Sweating induced by exercise or sauna, collected directly into bottle	17 participants with As detected in all samples Blood plasma mean: 2.5 (range 0.9–13) (*n* = 17) Urine mean: 37 (range 4.8–200) (*n* = 20) Sweat mean: 3.1 (range 3.7–22) (*n* = 20)

**Table 2:** Studies of cadmium excretion in sweat.

Study	Country, participants	Study design and intervention	Key findings (concentrations *μ*g/L unless otherwise indicated)
Genuis et al., 2010 [3]	Canada 10 with chronic conditions 10 healthy	Simultaneous measurement of toxic trace elements in blood plasma, urine, and sweat Exercise or sauna Sweat collected directly into bottle	3 participants with cadmium detected in all samples Blood plasma mean: 0.03 (range 0.02–0.07) (*n* = 11) Urine mean: 0.28 (0.18–0.39) (*n* = 3) Sweat mean: 5.7 (0.36–36) (*n* = 18)

Omokhodion and Howard, 1994 [30]	UK 15 healthy participants	Sweat collected using modified arm bag (hand excluded) Participants exercised at room temperature	Cadmium detected in 13 sweat samples Mean 1.9 Range 1.1–3.1

Stauber and Florence, 1988 [28]	Australia 24 males 13 females taking oral contraceptives 26 females not taking oral contraceptives	Forearm sweat induced by pilocarpine iontophoresis and collected on a membrane filter	Males mean sweat cadmium 1.4 (range <0.5–10) Females not taking contraceptives 2.6 (<0.5–18) Females taking contraceptives 2.4 (<0.5–5.5)

Stauber and Florence, 1987 [22]	Australia 9 males 7 females taking oral contraceptives 6 not taking oral contraceptives (unclear overlap with 1988 participants)	Forearm sweat induced by pilocarpine iontophoresis and collected on a membrane filter	Cadmium not detected in sweat (0.5 detection limit) Mean blood cadmium 0.8

Robinson and Weiss, 1980 [31]	USA 28 males (university faculty members)	Exercise and shower preceded sauna for sweat collection. Sweat collected as drips from forehead or nose	Sweat cadmium (range 11–200) Urine cadmium (range ND–67) Sweat/urine ratio (range 1.0–16) No correlation between the concentrations in urine and sweat

Robinson and Weiss, 1980 [32] (companion to previous)	USA 2 males (university faculty members)	As previous, cadmium also measured in hair segments.	Daily excretion of cadmium estimated as follows: (i) 30 *μ*g/day in urine (ii) 120 *μ*g/day in sweat (iii) 0.2 *μ*g/day in hair Cadmium concentrations in hair and sweat were lower in one participant than the other

Cohn and Emmett, 1978 [33]	USA 6 males 3 females	Total body washdown and arm bag techniques	Mean concentration of cadmium in sweat > urine Arm bags yielded lower levels than whole body measurements

**Table 3:** Studies of lead excretion in sweat.

Study	Country, participants	Study design and intervention	Key findings (concentrations *μ*g/L unless otherwise indicated)
Genuis et al., 2010 [3]	Canada 10 with chronic health conditions 10 healthy	Analyses of blood plasma, urine, and sweat Sweating induced by exercise or sauna, collected directly into bottle	Sweat mean 31 (range 1.5–94) (*n* = 20) Blood plasma mean 0.12 (0.39–1.7) (*n* = 20) Urine mean 1.8 (0.91–7.5) (*n* = 20)

Omokhodion and Crockford, 1991 [34]	UK 2 participants	Blood, urine, and sweat lead measured before and following ingestion of lead chloride: 1 or 2 doses of lead chloride (20 mg PbCl_2_ total, in 1 or 2 divided doses).	Blood lead peaked at 4 h Sweat concentrations did not increase significantly (range 0–11) Blood concentration range 6–51 Urine concentration range 10–97 Arm sweat collections varied by more than 2-fold between arms at the same time on the same person

Omokhodion and Howard, 1991 [35]	Unidentified “tropics” 19 workers in a lead battery factory 8 controls (medical students)	Measured lead in sweat, blood, and urine simultaneously Sweating induced by exercising at room temperature. Sweat collected in arm bags.	Workers: (i) blood lead 13–36 (ii) urine lead 28–290 *μ*g/g creatinine (iii) sweat lead 72–260 Controls: (i) blood lead 90–120 (ii) urine lead 9–20 *μ*g/g creatinine (iii) sweat lead 9–30

Omokhodion and Crockford, 1991 [36]	UK 24 normal, healthy subjects	Measured lead in sweat, urine, blood, and saliva Sweat collected in arm bags, sitting in a hot chamber	(i) Blood lead 86 (range 60–140) (ii) Urine lead 18 *μ*g/g creatinine (range 7.7–44 *μ*g/g creatinine) (iii) Mean sweat lead 5.2 (2.5–13) (iv) Saliva lead 4.8 (2.5–10)

Parpaleĭ et al., 1991 [27] (in Russian—English abstract only)	Russia NR in abstract	NR in abstract	“… sauna increased excretion with sweat fluid of toxic substances [lead] that penetrated the body during work. Sauna is recommended.”

Lilley et al., 1988 [37]	Australia 9 lead workers volunteers had lead applied to skin	Lead dust 6 h/day for 4 days 20 mg Pb dust on L arm of volunteer PbNO_3_ 24 h of 60 mg PbNO_3_ on L arm of volunteer.	Sweat lead in workers: 71–18,000 Following exposure, sweat lead from R arm increased approximately by 10x, returning to baseline after approximately by 2–4 days. Saliva increased approximately 5-6x. Urine and blood levels were unchanged

Stauber and Florence, 1988 [28]	Australia 24 males 13 females taking oral contraceptives 26 not taking oral contraceptives	Sweating induced on the forearms by pilocarpine iontophoresis and collected on a membrane filter	Mean sweat lead: (i) males: 41 (range 6–87) (ii) females not taking contraceptives: 24 (<5–66) (difference with males *P* < 0.01) (iii) females taking contraceptives: 36 (<5–70)

Stauber and Florence, 1987 [22]	Australia 9 males 7 females taking oral contraceptives 6 not taking oral contraceptives (unclear overlap with 1988 participants)	Sweating induced in the forearms by pilocarpine iontophoresis and collected on a membrane filter	No significant differences among groups Mean blood lead 200 Mean blood plasma lead 10 Mean sweat lead 15

Haber et al., 1985 [26] (in German-used extended abstract)	Germany 4 groups of 8 males 2 groups with occupational lead exposure 2 control groups	Comparison of precisely defined physical work (intensive cycling and extended rowing in a pool), examining lead excretion in persons with elevated blood levels compared with nonexposed controls	Aerobic endurance training (rowing) caused a significant drop in the blood lead level in the occupationally exposed group (mean 430 (range 320–580) decreased to 370 (240–450)) (*P* < 0.05) Endurance training was more effective than shorter, more intensive training (cycling) Urine lead levels were not significantly affected by training

Cohn and Emmett, 1978 [33]	USA 6 males 3 females	Total body washdown and arm bag techniques	The mean concentration of lead in sweat was similar to that in urine (1) Total body sweat lead mean: (i) males: 24 (SD 16) (ii) females: 53 (range 40–60) (2) Body minus 1 arm/arm bag sweat lead 60 (SD 16) (40–120)/83 (86) (20–250)

Hohnandel et al., 1973 [38]	33 healthy males 15 females	15 min of arm bag collection	Mean sweat lead: (i) males: 51 (range 8–180) (ii) females: 120 (SD 72) (49–280)

**Table 4:** Studies of mercury excretion in sweat.

Study	Country, participants	Study design and intervention	Key findings (concentrations *μ*g/L unless otherwise indicated)
Genuis et al., 2010 [3]	Canada 10 with chronic conditions 10 healthy	Sweating induced by exercise or sauna, collected directly into bottle	16 participants had mercury detected in all samples Blood plasma mercury mean 0.61 (range 0.26–1.6) (*n* = 16) Urine mean 0.65 (range 0.32–1.3) (*n* = 16) Sweat mean 0.86 (range 0.48–1.5) (*n* = 20)

Robinson and Skelly, 1983 [39]	USA 21 males at university 7 sampled more than once	Mercury in sweat dripping from forehead or nose, compared with urine	Sweat mean 0.5 (range 0.1–1.4)

Sunderman 1978 [40]	USA 1 case with mercury intoxication	Case report of chelating agents to treat mercury intoxication, followed by a regimen of daily sweat and physiotherapy for a protracted period of several months	Appreciable quantities of mercury were excreted in sweat. With the sweating regimen mercury, levels in sweat decreased to within the normal range

Lovejoy et al., 1973 [41]	USA 3 mercury-exposed workers 3 nonexposed workers 1 control	Participants wore rubber chest waders from 7 : 30 to 9 : 00 am Sweat accumulated in the feet was collected, as well as a 16-hour urine sample	Exposed workers: 1.5 h sweat: 120–350 ng mercury 16 h urine: 160–190 ng mercury Unexposed workers: 1.5 h sweat: 5–8 ng mercury 16 h urine: 5–7 ng mercury Internal controls: 1.5 h sweat: 43–70 ng mercury 16 h urine: 30–46 ng mercury Mercury concentrations in sweat > urine for exposed workers; similar for controls

## References

[1] Institute for Functional Medicine (2010). *Textbook of Functional Medicine*.

[2] Sears ME, Genuis SJ (2012). Environmental determinants of chronic disease, and medical approaches: recognition, avoidance, supportive therapy and detoxification. *Journal of Environmental and Public Health*.

[3] Genuis SJ, Birkholz D, Rodushkin I, Beesoon S (2011). Blood, urine, and sweat (BUS) study: monitoring and elimination of bioaccumulated toxic elements. *Archives of Environmental Contamination and Toxicology*.

[4] http://www.atsdr.cdc.gov/ToxProfiles/TP.asp?id=22&tid=3.

[5] http://www.atsdr.cdc.gov/toxprofiles/tp.asp?id=48&tid=15.

[6] http://www.atsdr.cdc.gov/ToxProfiles/tp.asp?id=96&tid=22.

[7] http://www.atsdr.cdc.gov/ToxProfiles/TP.asp?id=115&tid=24.

[8] Copes R, Clark NA, Rideout K, Palaty J, Teschke K (2008). Uptake of cadmium from Pacific oysters (*Crassostrea gigas*) in British Columbia oyster growers. *Environmental Research*.

[9] Perilli P, Mitchell LG, Grant CA, Pisantea M (2010). Cadmium concentration in durum wheat grain (*Triticum turgidum*) as influenced by nitrogen rate, seeding date and soil type. *Journal of the Science of Food and Agriculture*.

[10] Liu W, Zhou Q, An J, Sun Y, Liu R (2010). Variations in cadmium accumulation among Chinese cabbage cultivars and screening for Cd-safe cultivars. *Journal of Hazardous Materials*.

[11] Houston MC (2011). Role of mercury toxicity in hypertension, cardiovascular disease, and stroke. *Journal of Clinical Hypertension*.

[12] http://www.hc-sc.gc.ca/ewh-semt/pubs/contaminants/dhhssrl-rpecscepsh/index-eng.php.

[13] http://www.atsdr.cdc.gov/interactionprofiles/index.asp.

[14] Leppaluoto J (1988). Human thermoregulation in sauna. *Annals of Clinical Research*.

[15] Hannuksela ML, Ellahham S (2001). Benefits and risks of sauna bathing. *American Journal of Medicine*.

[16] Eisalo A, Luurila OJ (1988). The Finnish sauna and cardiovascular diseases. *Annals of Clinical Research*.

[17] Levisky JA, Bowerman DL, Jenkins WW, Karch SB (2000). Drug deposition in adipose tissue and skin: evidence for an alternative source of positive sweat patch tests. *Forensic Science International*.

[18] Cage GW, Dobson RL (1965). Sodium secretion and reabsorption in the human eccrine sweat gland. *Journal of Clinical Investigation*.

[19] Dill DB, Hall FG, van Beaumont W (1966). Sweat chloride concentration: sweat rate, metabolic rate, skin temperature, and age. *Journal of Applied Physiology*.

[20] Dill DB, Hall FG, Edwards HT (1938). Changes in composition of sweat during acclimatization to heat. *American Journal of Physiology*.

[21] Robinson S, Nicholas JR, Smith JH, Daly WJ, Pearcy M (1955). Time relation of renal and sweat gland adjustments to salt deficiency in men. *Journal of Applied Physiology*.

[22] Stauber JL, Florence TM (1987). The determination of trace metals in sweat by anodic stripping voltammetry. *Science of the Total Environment*.

[23] Montain SJ, Cheuvront SN, Lukaski HC (2007). Sweat mineral-element responses during 7 h of exercise-heat stress. *International Journal of Sport Nutrition and Exercise Metabolism*.

[24] Falk B, Bar-Or O, Calvert R, MacDougall JD (1992). Sweat gland response to exercise in the heat among pre-, mid-, and late- pubertal boys. *Medicine and Science in Sports and Exercise*.

[25] Falk B, Dotan R (2008). Children’s thermoregulation during exercise in the heat—a revisit. *Applied Physiology, Nutrition and Metabolism*.

[26] Haber P, Ring F, Jahn O, Meisinger V (1985). Influence of intensive and extensive aerobic circulatory stress on blood lead levels. *Zentralblatt für Arbeitsmedizin, Arbeitsschutz, Prophylaxe und Ergonomie*.

[27] Parpaleĭ IA, Prokof’eva LG, Obertas VG (1991). The use of the sauna for disease prevention in the workers of enterprises with chemical and physical occupational hazards. *Vracebnoe Delo Kiev*.

[28] Stauber JL, Florence TM (1988). A comparative study of copper, lead, cadmium and zinc in human sweat and blood. *Science of the Total Environment*.

[29] Yousuf AKM, Misbahuddin M, Rahman MS (2011). Secretion of arsenic, cholesterol, vitamin E, and zinc from the site of arsenical melanosis and leucomelanosis in skin. *Clinical Toxicology*.

[30] Omokhodion FO, Howard JM (1994). Trace elements in the sweat of acclimatized persons. *Clinica Chimica Acta*.

[31] Robinson JW, Weiss S (1980). The direct determination of cadmium in urine and perspiration using a carbon bed atomizer for atomic absorption spectroscopy. *Journal of Environmental Science and Health, Part A*.

[32] Robinson JW, Weiss S (1980). The direct determination of cadmium in hair using carbon bed atomic absorption spectroscopy. Daily rate of loss of cadmium in hair, urine and sweat. *Journal of Environmental Science and Health, Part A*.

[33] Cohn JR, Emmett EA (1978). The excretion of trace metals in human sweat. *Annals of Clinical and Laboratory Science*.

[34] Omokhodion FO, Crockford GW (1991). Sweat lead levels in persons with high blood lead levels: experimental elevation of blood lead by ingestion of lead chloride. *Science of the Total Environment*.

[35] Omokhodion FO, Howard JM (1991). Sweat lead levels in persons with high blood lead levels: lead in sweat of lead workers in the tropics. *Science of the Total Environment*.

[36] Omokhodion FO, Crockford GW (1991). Lead in sweat and its relationship to salivary and urinary levels in normal healthy subjects. *Science of the Total Environment*.

[37] Lilley SG, Florence TM, Stauber JL (1988). The use of sweat to monitor lead absorption through the skin. *Science of the Total Environment*.

[38] Hohnadel DC, Sunderman FW, Nechay MW, McNeely MD (1973). Atomic absorption spectrometry of nickel, copper, zinc, and lead in sweat collected from healthy subjects during sauna bathing. *Clinical Chemistry*.

[39] Robinson JW, Skelly EM (1983). The direct determination of mercury in sweat. *Spectroscopy Letters*.

[40] Sunderman FW (1978). Clinical response to therapeutic agents in poisoning from mercury vapor. *Annals of Clinical and Laboratory Science*.

[41] Lovejoy HB, Bell ZG, Vizena TR (1973). Mercury exposure evaluations and their correlation with urine mercury excretions. 4. Elimination of mercury by sweating. *Journal of Occupational Medicine*.

[42] Airey D (1983). Mercury in human hair due to environment and diet: a review. *Environmental Health Perspectives*.

[43] Stauber JL, Florence TM, Gulson BL, Dale LS (1994). Percutaneous absorption of inorganic lead compounds. *Science of the Total Environment*.

[44] Sällsten G, Kreku S, Unosson H (2000). A small dose of ethanol increases the exhalation of mercury in low-level-exposed humans. *Journal of Toxicology and Environmental Health, Part A*.

[45] Brown KW (2001). Workers’ health and colonial mercury mining at Huancavelica, Peru. *The Americas*.

[46] Kukkonen-Harjula K, Kauppinen K (2006). Health effects and risks of sauna bathing. *International Journal of Circumpolar Health*.

[47] Dahlgren J, Cecchini M, Takhar H, Paepke O (2007). Persistent organic pollutants in 9/11 world trade center rescue workers: reduction following detoxification. *Chemosphere*.

[48] Genuis SJ, Birkholz D, Ralitsch M, Thibault N (2010). Human detoxification of perfluorinated compounds. *Public Health*.

[49] Genuis SJ, Beesoon S, Birkholz D, Lobo RA (2012). Human excretion of bisphenol-A: blood, urine and sweat (BUS) study. *Journal of Environmental and Public Health*.

[50] Ross GH, Sternquist MC Methamphetamine exposure and chronic illness in police officers: significant improvement with sauna-based detoxification therapy.

[51] Weschler LB (2008). Sweat electrolyte concentrations obtained from within occlusive coverings are falsely high because sweat itself leaches skin electrolytes. *Journal of Applied Physiology*.

[52] Armstrong LE (2008). Commentary on viewpoint: sweat electrolyte concentrations obtained from within occlusive coverings are falsely high because sweat itself leaches skin electrolytes. *Journal of Applied Physiology*.

